# Immune‐mediated polyarthritis and anterior uveitis secondary to zonisamide administration in a dog with refractory epilepsy

**DOI:** 10.1002/vms3.1374

**Published:** 2024-02-25

**Authors:** Paula Baya, Saya Press, Stephanie Istvan, Kaila Rizzo

**Affiliations:** ^1^ Department of Emergency and Critical Care, Veterinary Specialty Hospital of San Diego Ethos Veterinary Health San Diego California USA

**Keywords:** adverse reactions, epilepsy, polyarthritis, uveitis, zonisamide

## Abstract

The objective of this article is to describe a case of suspected zonisamide‐induced immune‐mediated polyarthritis (IMPA) and anterior uveitis in a dog. A 7‐year‐old male neutered Siberian Husky with a history of refractory idiopathic epilepsy was presented for cluster seizures. Following the addition of zonisamide to the antiepileptic regime, the dog developed new IMPA and anterior uveitis. Within a few weeks of discontinuation of the zonisamide, the dog's IMPA and anterior uveitis resolved. These immune‐mediated conditions were thus presumed to be an idiosyncratic reaction to zonisamide. To our knowledge, this is the first report of IMPA and anterior uveitis in dogs associated with zonisamide administration at its recommended dose.

## Graphical abstract

A dog with refractory seizures was presumed to have developed immune‐mediated polyarthritis (IMPA) and anterior uveitis secondary to zonisamide administration. Symptoms began shortly after the initiation of zonisamide and completely resolved within 2 weeks of discontinuation. Diagnostics did not reveal any other causes.

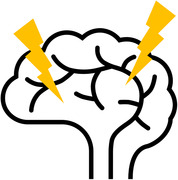

## 1 INTRODUCTION

Zonisamide is a 1,2‐benzisoxazole‐3‐methanesulfonamide antiepileptic drug used in veterinary and human medicine (Dewey et al., 2004; Fukunaga et al., 2009; Schwartz et al., 2011). It is a benzisoxazole sulphonamide derivative, which unlike other common sulphonamide‐based drugs, lacks a non‐arylamine sulphonamide group. The significance of the differing sulphonamide component in zonisamide and its role in adverse effects remains unknown (Fukunaga et al., 2009; Kanazono et al., 2021). Zonisamide is available in injectable and oral formulations with therapeutic dosages in dogs ranging from 5 to 15 mg/kg every 12 h (Brewer et al., 2014; Fukunaga et al., 2009; Kanazono et al., 2021; Podell et al., 2016). In veterinary medicine, zonisamide is considered an adjunct therapy and is infrequently used as a monotherapy for epilepsy management (Boothe & Perkins, 2008; Brewer et al., 2014; Podell et al., 2016; Schwartz et al., 2011). The exact mechanism of action of the drug is unconfirmed; however, it is theorized to work on the sodium and T‐type calcium channels to inhibit hypersynchronization at neuronal membranes and prevent progression of seizures from cortex to subcortex (Boothe & Perkins, 2008; Chung et al., 2012; Dewey et al., 2004; Schwartz et al., 2011). Additionally, zonisamide may directly affect neurotransmitters (glutamate, gamma aminobutyric acid, dopamine, serotonin and acetylcholine) and have neuroprotective properties, though the clinical significance of this is unknown (Schwartz et al., 2011). Zonisamide is also a carbon anhydrase inhibitor. This activity does not contribute to antiepileptic management (Cook et al., 2011; Dewey et al., 2004). In people, its use extends to the treatment of other neurological conditions such as migraines, intention tremors, neuropathic pain and Parkinson's disease (Kanazono et al., 2021).

Reported adverse effects of zonisamide in dogs include sedation, ataxia, vomiting, decreased thyroxine (T4) and hepatopathy (Kanazono et al., 2021; Klopmann et al., 2007; Miller et al., 2011; Schwartz et al., 2011; Twedt et al., 1997). There are case reports of dogs developing hepatic necrosis, acute tubular acidosis, erythema multiforme and behavioural changes such as aggression (Cook et al., 2011; Kanazono et al., 2021; Klopmann et al., 2007; Miller et al., 2011; Podell et al., 2016; Safadi et al., 2021; Schwartz et al., 2011; Twedt et al., 1997). In three dogs, aggression resolved with discontinuation of zonisamide and relapsed with re‐exposure to zonisamide (Kanazono et al., 2021). In people, rare cognitive and psychiatric disturbances have been described; however, adverse effects appear to be dose dependent and typically include dizziness, hyporexia, nausea and somnolence (Charalambous et al., 2016; Kanazono et al., 2021; Miller et al., 2011). The purpose of this report is to describe the development of immune mediated polyarthritis (IMPA) and anterior uveitis presumed to be secondary to zonisamide administration in a dog. To the authors’ knowledge, this is the first description of IMPA and anterior uveitis as adverse effects of zonisamide.

## 2 CASE SUMMARY

A 7‐year‐old, 33.9 kg male neutered Siberian Husky was presented to the emergency service after having three generalized, tonic–clonic seizures within 24 h. The dog had two seizures in close succession, prompting the administration of a rescue protocol at home which consisted of midazolam (Westward/Hikma; 0.07 mg/kg intranasally once), clorazepate^1^ (0.44 mg/kg, PO, once) and levetiracetam extended‐release (ER) (Keppra; Zhejiang Huahai Pharmaceutical; 22.1 mg/kg, PO, once). An additional seizure occurred a few hours later, and the dog was admitted shortly afterwards. The dog was already being treated for presumptive idiopathic epilepsy and for the 7 months had been receiving phenobarbital (e5 Pharma, LLC; 3.8 mg/kg, PO, q12h), potassium bromide (KBro Vet; Pegasus Laboratories; 10.9 mg/kg, PO, q12h) and levetiracetam ER (22.1 mg/kg, PO, q12h) as well as Purina Neurocare dry kibble (Purina ProPlan Veterinary Diets; Nestlé Purina PetCare Company). Other medications included trazodone (Teva; 2.9 mg/kg, PO, PRN), Visbiome^2^ 1 capsule (450bcfu, PO, q24h), phenylpropanolamine (Proin; Pegasus Laboratories; dose unknown) and milk thistle + *S*‐adenosylmethionine (Denamarin; Nutramax Laboratories; 6.6 mg/kg, PO, every 14 days). The dog had a prior history of seizures that had started as a puppy, polyuria and several unrelated episodes of pneumonia; however, the details of any previous management were unknown due to recent adoption.

At presentation, routine physical and neurologic examinations, vital parameters and venous blood gas were normal. The serum phenobarbital level was within the therapeutic interval (26 μg/mL, [reference interval [RI]: 15–35 μg/mL]). In the 6 months leading to this event, seizure management had been challenging with an increased frequency of breakthrough seizures. Low phenobarbital levels (<15 μg/mL) and persistently low bromide levels (<1.0 mg/mL) had required frequent medication adjustments.

The dog was admitted to the ICU for 10 h for seizure monitoring and received levetiracetam ER (38.4 mg/kg, PO, once). The levetiracetam ER dose was increased to 38.4 mg/kg, PO, q12h. The patient was discharged after being seizure‐free with a normal neurologic exam but returned a few hours following discharge after having two seizures with an extended post‐ictal phase. At re‐presentation, the dog was non‐ambulatory, which improved to ambulation with ataxia by the time of admission to the ICU approximately 2 h later. The remainder of the neurologic examination was normal. The dog was hospitalized for monitoring, and zonisamide (Glenmark; 3.0 mg/kg, PO, q12h) was added to the treatment regime. After 24 h without seizures the potassium bromide dose was increased to 13.1 mg/kg, PO, q12h and the zonisamide dose was doubled to 6.0 mg/kg, PO, q12h. The levetiracetam and phenobarbital were kept at the previously prescribed doses. The dog remained seizure‐free for an additional 24 h and was then discharged.

Following discharge, no further seizures were reported, but the dog re‐presented 6 days later for anorexia, and an acute onset of reluctance to ambulate. Physical examination identified pyrexia (41.3°C [106.4°F]), weak ambulation with generalized ataxia, joint effusion in all joints, erythema and pitting oedema in the right pelvic limb, dull mentation, abdominal pain, ocular changes (bilateral blepharospasm, mucopurulent ocular discharge, conjunctivitis and mucopurulent nasal discharge) and a newly auscultated grade I/VI left apical systolic heart murmur. Point of care thoracic and abdominal ultrasound (Sonoscape Medical Corp) were unremarkable and in‐house serum testing for, *Dirofilaria immitis* antigen and for *Anaplasma phagocytophilum, Anaplasma platys, Borrelia burgdorferi, Ehrlichia canis* and *Ehrlichia ewingii* antibodies (IDEXX SNAP 4Dx test, IDEXX Laboratories Inc) was negative. A conjunctival swab was submitted externally for a canine infectious respiratory disease PCR panel (Canine Respiratory PCR; IDEXX External Reference Laboratories). Thoracic radiographs demonstrated a focal interstitial pattern in the left cranial lung lobe and a ventrally distributed unstructured interstitial pulmonary pattern coalescing to alveolar in the right middle lung lobe, with concern for pulmonary fibrosis or early aspiration pneumonia. A complete blood count (CBC) (Zoetis Reference Lab San Diego) revealed an inflammatory leukogram (WBC 19200/μL [RI: 4400–14,600/μL], neutrophils 17,110/μL [RI: 2394–7514/μL], monocytes 1150/μL [RI: 88–1094/μL]), and a serum biochemistry profile (Zoetis Reference Lab San Diego) indicated mild hypoalbuminemia (2.5 g/dL [RI: 2.7–3.9 g/dL]), mild hyperglobulinemia (4.1 g/dL [RI: 2.2–3.7 g/dL]), elevated alkaline phosphatase (1122 U/L [RI: 8–196 U/L]), hyperbilirubinemia (0.4 mg/dL [RI: 0.0–0.3 mg/dL]) and elevated cholesterol (397 mg/dL [RI: 131–346 mg/dL]). Medical interventions included Lactated Ringer's IV fluids (ICU Medical, Inc.) (120 mL/h), methadone (Mylan, LLC; 0.05 mg/kg, IV, PRN), maropitant (Cerenia; Zoetis; 1 mg/kg, IV, q24h), ampicillin sulbactam (Unasyn; Pfizer Roering; 30 mg/kg, IV, q8h) and continuation of the previously prescribed antiepileptic drugs (phenobarbital, potassium bromide, levetiracetam ER, zonisamide).

The dog was hospitalized overnight, during which time the rectal temperature improved to 39.5°C (103.1°F), but all other clinical signs remained static. The following day, serum was submitted for fungal antibody titres (Zoetis Reference Lab San Diego; *Histoplasma capsulatum, Cryptococcus neoformans*, and *Coccidioides immitis*) and arthrocentesis of the right stifle, right carpus and left elbow were performed. In‐house synovial fluid cytology (Zoetis Reference Lab San Diego) from all three joints identified neutrophilic inflammation consistent with IMPA. The samples were also submitted externally for assessment by a clinical pathologist. Urinalysis (Zoetis Reference Lab San Diego) indicated isosthenuria (urine specific gravity 1.012 [RI: 1.020–1.045]) and a quiet sediment. Ocular testing revealed decreased intraocular pressure OU (OD 8 mmHg and OS 6 mmHg [RI: 15–25 mmHg]), decreased tear production OU (no reported value), negative fluorescein stain uptake and normal fundic exam OU. The treatment plan was adjusted to treat the presumed IMPA and anterior uveitis. Suspicion of an atypical reaction to zonisamide prompted the decision to discontinue this antiepileptic. Treatments included eye lubrication (Aventix; 0.25 in. strip, OU, q8h), prednisolone acetate (Alon Laboratories; 1 drop, OU, q12h), 0.2% cyclosporine ointment (Merck Animal Health; 0.25 in. strip, OU, q12h), doxycycline (Cadilla; 4.8 mg/kg, PO, q12h), enrofloxacin (Baytril; Bayer/Elanco; 14 mg/kg, IV, q24h) and dexamethasone sodium phosphate (Bimeda MTC Animal Health; 0.1 mg/kg, IV, q24h). Other changes in therapy included transitioning from maropitant to ondansetron (Intas Pharmaceuticals Limited; 0.3 mg/kg, IV, q8h) due to cost, increasing the methadone dose (0.1 mg/kg, IV, q6h), and decreasing the fluid rate to 80 mL/h. Ampicillin sulbactam was replaced with doxycycline and enrofloxacin due to concerns regarding the possibility of tickborne disease. Serology for tickborne diseases was offered to client but was declined. The external synovial fluid analysis results were still pending, and septic inflammation remained a possibility.

Twenty‐four hours following admission, venous blood gas analysis (Epocal INC) remained unremarkable. A CBC and serum chemistry indicated mild progression of the inflammatory leukogram (WBC 25,500/μL, neutrophils 21,692/μL, monocytes 2297/μL), hypoalbuminemia (2.2 g/dL), hyperglobulinemia (4.1 g/dL), elevated alkaline phosphatase (1220 U/L), hyperbilirubinemia (0.4 mg/dL) and elevated cholesterol (420 mg/dL). The dog was clinically static on day 2, and treatment remained the same. On day 3 of hospitalization (48 h after zonisamide was discontinued), the dog's mentation, pain, intraocular pressures, blepharospasm and pitting oedema had improved. The decreased tear production and the heart murmur were persistent. Additionally, the dog developed polyuria, possibly secondary to intravenous fluid administration, steroid therapy and/or historic polyuria. The dog's bodyweight reduced from 33.9 kg at admission to 31.4 kg.

On day 4 of hospitalization, the dog was transitioned from dexamethasone sodium phosphate to prednisone (Jubilant Cadista; 1.2 mg/kg, PO, q24h) and from methadone to gabapentin (Markasas Pharm Ltd; 9.5 mg/kg, PO, q8h). Ophthalmologic evaluation by a board‐certified ophthalmologist revealed bilateral corneal oedema, punctate intra‐retinal haemorrhage, improving intraocular pressures (OD 9 mmHg, OS 8 mmHg) and resolving anterior uveitis. Apart from the ocular abnormalities and polyuria and polydipsia, all other reported clinical signs had resolved. Results of canine infectious respiratory PCR testing (canine distemper virus RealPCR, *Bordetella bronchiseptica* RealPCR, canine adenovirus type 2 RealPCR, canine herpesvirus type 1 RealPCR, canine parainfluenza virus RealPCR, canine respiratory coronavirus RealPCR, H3N2 influenza virus RealPCR, influenza A virus RealPCR, *Mycoplasma cynos* RealPCR, *Streptococcus equi* subsp. *Streptococcus zooepidemicus* RealPCR, canine pneumovirus RealPCR) and serum fungal antibody titres (*H. capsulatum, C. neoformans, Cocci‐IgM, Cocci‐IgG*) returned prior to discharge and were negative (Tables 1 and 2). The dog was discharged at the end of day 4 (72 h after zonisamide was discontinued) with gabapentin (9.6 mg/kg, PO, q8–12h), enrofloxacin (8.7 mg/kg, PO, q24h), doxycycline (3.2 mg/kg, PO, q12h), prednisone (0.96 mg/kg, PO, q24h), prednisolone acetate ophthalmic solution 1% (1 drop, OU, q12h) and 0.2% cyclosporine ointment (0.25 in. strip, OU, q12h) in addition to the antiepileptic medications (phenobarbital, potassium bromide and levetiracetam ER). The doses for antiepileptic therapy were as follows: phenobarbital (4.1 mg/kg, PO, q12h), potassium bromide (13.9 mg/kg, PO, q12h) and levetiracetam ER (39.8 mg/kg, PO, q12h). Antibiotics were discontinued 3 days later following the absence of pathogens found on urinary culture (IDEXX External Reference Laboratories) and synovial cytology. External synovial fluid cytology confirmed a cytologic diagnosis of IMPA (>3000/μL nucleated cells; 97% mature non‐degenerative neutrophils; no infectious or neoplastic agents present).

**TABLE 1:** The findings of the canine respiratory disease RealPCR comprehensive panel^l^ from a conjunctival swab.

Test	Result
Canine distemper virus RealPCR	Negative
*Bordetella bronchiseptica* RealPCR	Negative
Canine adenovirus type 2 RealPCR	Negative
Canine herpesvirus type 1 RealPCR	Negative
Canine parainfluenza virus RealPCR	Negative
Canine respiratory coronavirus RealPCR	Negative
H3N2 influenza virus RealPCR	Negative
Influenza A virus RealPCR	Negative
*Mycoplasma cynos* RealPCR	Negative
*Streptococcus equi* subsp. *Streptococcus zooepidemicus* RealPCR	Negative
Canine pneumovirus RealPCR	Negative

**TABLE 2:** The results of fungal serologic testing^s^.

Test	Antibody Interpretation	Result
*Histoplasma capsulatum*	<1:1	Negative
*Cryptococcus neoformans*	<1:2	Negative
Cocci‐IgM	<1:1	Negative
Cocci‐IgG	<1:1	Negative

The dog returned 2 weeks later for a recheck of CBC, serum biochemistry (Table 3) anterior uveitis and IMPA. The only notable abnormality on physical examination was improved but low normal ocular pressures (OD 16 mmHg, OS 14 mmHg [RI: 15–25 mmHg]). The previously reported joint effusion had been resolved. Ocular prednisolone acetate drops were discontinued, and the oral prednisone was tapered over 10 days before being discontinued. No additional seizures were reported at home. The dog returned 6 weeks after discontinuation of zonisamide (27 days after discontinuation of steroids) for re‐examination and for assessment of serum potassium bromide level, at which time no recurrence of uveitis or joint effusion was reported. The dog remained seizure‐free.

**TABLE 3:** Serial bloodwork highlighting key findings from complete blood cell count^m^ and serum biochemistry^n^ from day 1 hospitalization, day 2 hospitalization and 10 days post‐discharge.

Serial bloodwork				
Test parameter	Day 1	Day 2	Recheck (10 days after discharge)	Reference interval
White blood cell (WBC)	19,200/μL	25,500/μL	12,500/μL	4400–14,600/μL
Neutrophils	17,110/μL	21,692/μL	9333/μL	2394–7514/μL
Monocytes	1150/μL	2297/μL	598/μL	88–1094/μL
Albumin	2.5 g/dL	2.2 g/dL	3.3 g/dL	2.7–3.9 g/dL
Globulin	4.1 g/dL	4.1 g/dL	3.6 g/dL	2.2–3.7 g/dL
Alkaline phosphatase	1122 U/L	1220 U/L	1586 U/L	8–196 U/L
Bilirubin	0.4 mg/dL	0.4 mg/dL	0.1 mg/dL	0.0–0.3 mg/dL
Cholesterol	397 mg/dL	420 mg/dL	299 mg/dL	131–346 mg/dL

## 3 DISCUSSION

This case report is, to the authors’ knowledge, the first description of anterior uveitis and IMPA suspected to be secondary to zonisamide treatment. The presumptive diagnosis was based on the clinical signs, history, exclusion of other concurrent disorders and resolution of clinical signs following the discontinuation of zonisamide.

A four‐type classification system for the description of drug reactions has been adapted for veterinary medicine based on the human system (Podell et al., 2016). Reported adverse effects for zonisamide include type I dose‐dependent side effects related to the pharmacology of the medication, such as sedation, ataxia, vomiting and anorexia (Charalambous et al., 2016; Cook et al., 2011; Miller et al., 2011; Podell et al., 2016; Schwartz et al., 2011). Type II idiosyncratic effects include renal tubular acidosis, systemic lupus erythematosus, photosensitive lichenoid drug eruption, toxic epidermal necrosis and acute hepatic necrosis (Charalambous et al., 2016; Cook et al., 2011; Miller et al., 2011; Podell et al., 2016; Safadi et al., 2021; Schwartz et al., 2011; Twedt et al., 1997). Type III adverse reactions are cumulative effects of long‐term treatment that include hypoalbuminemia, hypoproteinemia, decreased total T4, albumin and increased liver enzymes (Kanazono et al., 2021; Podell et al., 2016). Type IV delayed adverse effects have never been reported with zonisamide (Podell et al., 2016).

**FIGURE 1:**
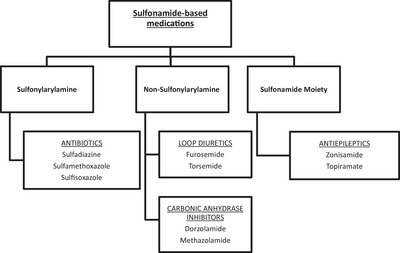
Classifications of sulphonamide drugs focussing specifically on Sulfonylarylamine, Non‐Sulfonylarylamine and Sulfonamide Moiety. Sulfonylarylamine class include antibiotics. Non‐Sulfonylarylamine class includes loop diuretics and carbonic anhydrase inhibitors. Sulfonamide Moiety class includes antiepileptics (Ghimire et al., 2013; Madisson et al., 2008; Ovung & Bhattacharyya, 2021; Verdel et al., 2006).^3^

**FIGURE 2:**
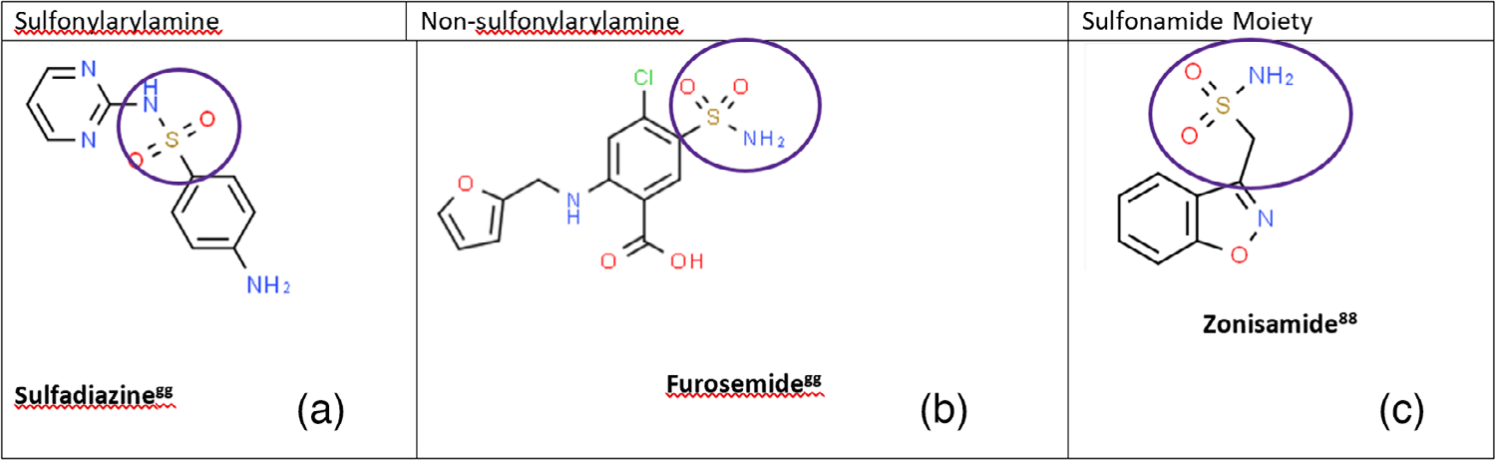
Classification of sulphonamide drugs and structural differences between the classes with focus on sulphonamide structure. Purple circle denotes position of sulphonamide structure in each class. (a) *Sulfonylarylamines*: In this group of drugs, which includes sulphadiazine, the sulphonamide chemical structure is attached directly to the benzene ring via an amine group (‐NH2) at the N4 position. (b) *Non‐sulfonylarylamines*: The drugs in this class feature a sulphonamide chemical structure attached to a benzene ring with amine group attached to the cyclic structure, rather in the N4 position. An example of a drug in this class is furosemide. (c) *Sulfonamide moiety*: Zonisamide, like other drugs in this class, features a sulphonamide chemical structure that is not connected directly to any cyclic structure (Ghimire et al., 2013; Madisson et al., 2008; Ovung & Bhattacharyya, 2021; Verdel et al., 2006).

The mechanism of zonisamide‐induced IMPA in this case is unknown. The sulphonamide group in zonisamide's chemical structure may play a role in stimulating immunogenic reactions, as suggested by prior reports documenting drug reactions to sulphonamide antimicrobials (Ackermann et al., 2015; Ghimire et al., 2013). Sulphonamide antimicrobials containing sulphamethoxazole and sulphadiazine have resulted in type I reactions that include keratoconjunctivitis sicca (KCS) and idiosyncratic reactions that include fever, acute hepatopathy and non‐septic polyarthritis in dogs (Ackermann et al., 2015; Charalambous et al., 2016; Dewey et al., 2004; Schwartz et al., 2011). A 2011 study describes a lethal subacute^3^ idiosyncratic hepatic necrosis following zonisamide administration to a dog experiencing refractory idiopathic seizures (Miller et al., 2011). The authors of that study report similar clinical presentations between the dogs in the study and people with sulphonamide antibiotic toxicity. Although controversial, multiple case reports in people have suggested cross‐reactivity between sulphonamide drug classes (Figure 1a–c) including non‐antimicrobials (Charalambous et al., 2016; Dewey et al., 2004; Podell et al., 2016; Schwartz et al., 2011; Verdel et al., 2006). Given the similarities in the chemical structures of sulphonamide antimicrobials and sulphonamide non‐antimicrobials (Figure 2), cross‐reactivity could potentially explain the similarities in adverse effects (Podell et al., 2016; Schwartz et al., 2011; Strom et al., 2003; Verdel et al., 2006).

In people, sulphonamides are a common drug class associated with allergic reactions, but evidence varies as to whether the sensitivity is due to the chemical structure or whether the patient has increased susceptibility to adverse reactions regardless of chemical structure (Khan & Chow, 2022; Khan et al., 2022; Strom et al., 2003; Verdel et al., 2006). A 2003 report describes that 9% of patients with a history of adverse reaction to sulphonamide antibiotics developed adverse reactions following administration of a non‐antibiotic sulphonamide medication (Khan et al., 2022; Strom et al., 2003). There is speculation that either T‐cell or IgE mediated responses to reactive metabolites play a role in sulphonamide antibiotic induced hypersensitivity. Further, there is consideration that the N4 amine ring drives the formation of reactive metabolites (Figure 2). The current consensus from the American Academy of Allergy, Asthma, and Immunology is that patients with a history of prior reaction to sulphonamide antibiotics are at a slightly increased risk for adverse reaction to sulphonamide non‐antibiotics. However, this is suspected to be secondary to a predisposition to overall drug hypersensitivities rather than immunologic driven cross‐reactivity. Multiple studies have shown that patients with sulphonamide hypersensitivities had a history of allergies to at least one other drug class (Khan et al., 2022). Sulphonamide allergy and cross‐reactivity continue to remain a topic of study in human medicine. To date, there are no studies in veterinary medicine assessing the hypersensitivity of non‐antimicrobial sulphonamides in patients with known sulphonamide antibiotic reactions. In this report, the dog had no known historic exposure to sulphonamide antibiotics; thus, predisposition to adverse reactions cannot be ruled out. However, with a reported history of pneumonia and a lack of prior medical records due to recent adoption, the authors cannot confirm this with certainty.

Studies highlighting sulphonamide antibiotic adverse effects in dogs include a report of one dog that developed non‐septic polyarthritis secondary to trimethoprim‐sulphonamide. This dog's signs were suspected to be due to trimethoprim‐sulphonamide because the signs improved in 1–3 days following withdrawal of medication and returned when the dog was re‐challenged with the medication (Trepanier et al., 2003). Similarly, a dog receiving zonisamide for idiopathic epilepsy developed polyarthropathy (Dewey et al., 2004). However, this dog also had high titres for Lyme disease, and the associated lameness resolved with doxycycline administration despite no changes in zonisamide dose. It is thus unclear if the use of zonisamide exacerbated the symptoms of Lyme polyathropathy, or if zonisamide had no impact on the dog's clinical picture (Dewey et al., 2004). In the same study of zonisamide use for idiopathic epilepsy, another dog developed transient KCS following zonisamide treatment that improved with topical cyclosporine despite continuation of zonisamide (Dewey et al., 2004). The authors could not identify other causes aside from the addition of zonisamide or compounding effect of multiple medications (Dewey et al., 2004).

In this report, the potential reasons for this dog's ocular pathology, including anterior uveitis and decreased tear production, are direct toxicity and immune‐mediated mechanisms (Trepanier et al., 2003). Sulphonamide‐induced KCS has been identified with sulphasalazine, which is cytotoxic to the lacrimal gland. With regards to an immune‐mediated mechanism, the presence of antibodies to platelets and T‐cells has been shown in dogs with sulphonamide hypersensitivity. The adaptive immune system has been implicated in the development of KCS and uveitis and may contribute to the ocular changes described in this study (Trepanier et al., 2003).

Another potential explanation for decreased tear production and uveitis could be zonisamide's carbonic anhydrase inhibitor activity (Beckwith‐Cohen et al., 2015; Cawrse et al., 2001). Carbonic anhydrase inhibitors, such as dorzolamide, are often used to manage glaucoma (Beckwith‐Cohen et al., 2015; Cawrse et al., 2001). Dorzolamide is a sulphonamide medication, and it should be used cautiously in dogs with sulphonamide hypersensitivities (Cawrse et al., 2001). Like zonisamide, dorzolamide's sulphonamide group is not attached to the benzene ring; however, despite this, it has been reported to cause idiosyncratic reactions similar to other sulphonamides (Cawrse et al., 2001; Ghimire et al., 2013; Trepanier et al., 2003). Several case reports in people document sulphonamide‐induced uveitis, more specifically non‐granulomatous anterior uveitis (Tilden, 1991). One study describes patients developing non‐granulomatous anterior uveitis within 1 week of initiating trimethoprim‐sulphamethoxazole (TMS), and after re‐exposure, clinical signs of uveitis developed within 24 h (Tilden, 1991). Some patients re‐challenged with a sulphonamide medication developed other sulphonamide‐induced adverse effects such as erythema multiforme, stomatitis and abnormal liver function (Tilden, 1991). All patients had resolution of anterior uveitis with discontinuation of TMS and topical steroid use (Tilden, 1991). The exact mechanism of sulphonamide‐induced uveitis is unknown as it is less frequently seen in comparison to other sulphonamide‐induced reactions in people. In this report, the dog was not re‐challenged with zonisamide to assess for recurrence of signs, so a definitive diagnosis of drug reaction cannot be confirmed. However, given the temporal association, drug class and lack of alternative explanation despite extensive investigations, there is strong suspicion that the uveitis was secondary to zonisamide. The similarities in the development and resolution of anterior uveitis in this case and previously documented sulphonamide‐induced anterior uveitis provide further support to this claim. To the authors’ knowledge, there have been no reports of zonisamide induced uveitis despite the reports of sulphonamide‐induced uveitis.

There are several limitations in this case report. The first is the administration of glucocorticoids. The dog experienced improvement after discontinuation of zonisamide; however, glucocorticoids were started concurrently raising the question of whether resolution of the clinical signs was purely due to discontinuation of zonisamide, or whether improvement would have occurred with glucocorticoids irrespective of zonisamide discontinuation. Nevertheless, in the case of an idiopathic immune‐mediated aetiology, long courses of glucocorticoid treatment are invariably required for successful treatment, so recurrence would have been expected following the very short 10‐day course used in this dog. A second limitation is the use of antibiotics. The administration of two doses of ampicillin sulbactam prior to sampling could have prevented the identification of bacteria on synovial fluid, and several days of subsequent treatment with doxycycline and enrofloxacin may have then eradicated an inciting infection. Moreover, synovial fluid cytology is not a sensitive test for the detection of infectious arthritis and synovial fluid cytology and PCR testing for infectious causes was not performed. It is therefore possible that resolution of clinical signs is correlated with effective infection control rather than discontinuation of zonisamide. Nevertheless, with the exception of tickborne diseases, infectious causes of polyarthritis are rare, and, considering the absence of reported tick exposure and negative serology results, the former is unlikely. The patient also received only a very short course of antibiotics, which would usually be considered insufficient for effective treatment of infectious polyarthritis. Third, the lack of an initial STT value makes it difficult to determine if poor tear production was significant enough to include KCS as a potential adverse effect of zonisamide. This could have provided further support for the cross‐reactivity of sulphonamide medications.

Most importantly, re‐challenge with zonisamide and recurrence of the clinical signs would be required to definitively prove the role of zonisamide. In a 2021 case report where dogs receiving zonisamide developed aggression, behaviour was improved and resolved within a week of discontinuing medication (Kanazono et al., 2021). Similarly, in a 2022 case series, four dogs developed febrile neutropenia, which began to resolve within a few days of discontinuing zonisamide (Brandifino et al., 2022). In both studies, the dog's symptoms improved, and extensive diagnostics, including bone marrow aspirates in the latter study, did not identify any other cause for symptoms (Brandifino et al., 2022; Kanazono et al., 2021). Many parallels can be drawn between these studies and the current study, particularly the improvement to resolution of clinical symptoms with the discontinuation of zonisamide. The symptoms in these two studies as well as the current study were presumed to be associated with zonisamide; thus, the medication was discontinued. Although supportive care was provided, alleviation of symptoms occurred sooner than anticipated, and no other underlying conditions were identified on diagnostics performed. The authors in the 2022 case series acknowledged that re‐challenge with zonisamide would definitively confirm hypothesis; however, there were humane concerns with re‐exposure (Brandifino et al., 2022). In the current study, as the dog's seizure control was adequate at follow‐up visits, reintroduction of additional antiseizure medications was not required. Additionally, given the severity of the dog's clinical symptoms, there were ethical concerns with re‐exposure.

## 4 CONCLUSION

In conclusion, this case report describes IMPA and anterior uveitis, presumed to be secondary to zonisamide. This complication has not been previously documented; however, other immune‐mediated and idiosyncratic reactions have been reported. Though more information is needed to fully understand potential reactions to zonisamide, this case report suggests that zonisamide use in dogs with known immune‐mediated diseases, hypersensitivities to sulphonamides or at risk of sulphonamide reaction should be monitored closely. At this time, sulfadiazine is the only sulpha drug in the United States listed with a warning for cross reactivity in people with sulphonamide hypersensitivity (Ghimire et al., 2013).

## AUTHOR CONTRIBUTIONS

*Conceptualization; data curation; formal analysis; investigation; resources; visualization; writing – original draft; writing – review and editing*: Paula Baya. *Conceptualization; formal analysis; resources; supervision; writing – review and editing*: Saya Press. *Conceptualization; formal analysis; resources; supervision; validation; writing – review and editing*: Stephanie Istvan. *Conceptualization; data curation; formal analysis; resources; supervision; writing – review and editing*: Kaila Rizzo.

## ETHICS STATEMENT

The authors confirm that the ethical policies of the journal, as noted on the journal's author guidelines page, have been adhered to. No ethical approval was required as this is a review article with no original research data.

### PEER REVIEW

The peer review history for this article is available at https://www.webofscience.com/api/gateway/wos/peer-review/10.1002/vms3.1374.

## Data Availability

Data sharing is not applicable to this article as no datasets were generated or analysed during the current study.

## Footnotes

1 Long‐term medication of owner acquired from different human pharmacies; unable to determine manufacturer.

2 It is the original De Simone probiotic formulation, previously sold under the name VSL#3 and is now manufactured exclusively by ExeGi Pharma.

3 Chemical structures provided in this image were generated from ChemSpider. ChemSpider is a free chemical structure database called that is owned by the Royal Society of Chemistry. Below are the citations associated with each chemical structure: https://www.chemspider.com/Chemical‐Structure.5532.html; https://www.chemspider.com/Chemical‐Structure.3322.html; https://www.chemspider.com/Chemical‐Structure.5026.html.
